# Comparative Gene Expression Analysis of *Salmonella* Typhimurium DT104 in Ground Chicken Extract and Brain Heart Infusion Broth

**DOI:** 10.3390/microorganisms12071461

**Published:** 2024-07-18

**Authors:** Yanhong Liu, Fangyuan Zhang, Jabari L. Hawkins, Jake R. Elder, Gian Marco Baranzoni, Zuyi Huang, Pina M. Fratamico, Salina Parveen

**Affiliations:** 1 Molecular Characterization of Foodborne Pathogens Research Unit, Eastern Regional Research Center, Agricultural Research Service, U.S. Department of Agriculture, 600 East Mermaid Lane, Wyndmoor, PA 19038, USAgmbaranzoni@gmail.com (G.M.B.);; 2 Department of Chemical and Biological Engineering, Villanova University, Villanova, PA 19085, USA; 3 Food and Agricultural Sciences Graduate Program, Food and Resource Sciences, U.S. Department of Agriculture, University of Maryland Eastern Shore, Princess Anne, MD 21853, USA

**Keywords:** *Salmonella enterica* Typhimurium DT104, poultry products, ground chicken extract, transcriptomics

## Abstract

*Salmonella enterica* Typhimurium DT104 (*S.* Typhimurium DT104) is an important foodborne pathogen that is associated with poultry and poultry products. Currently, there is very little information on the underlying molecular mechanisms that allow DT104 to survive and propagate in poultry meat and the poultry processing environment. The current study assessed the global gene expression of DT104 in ground chicken extract (GCE) compared to brain heart infusion (BHI) medium using RNA-Seq technology. DT104 was grown to the early stationary phase (ESP), inoculated into GCE or BHI, and then re-grown to the log phase before RNA was extracted and transcripts were quantified by RNA-Seq. Gene expression for DT104 grown in GCE was then compared to that of DT104 grown in BHI for samples grown to the ESP. Growth in GCE resulted in the up-regulated expression of genes related to translation, carnitine metabolism (23–283-fold change), and cobalamin (vitamin B12) biosynthesis (14-fold change). In particular, the presence of carnitine in chicken meat, and thus, in GCE, which lacks carbohydrates, may allow *Salmonella* to utilize this compound as a carbon and nitrogen source. This study demonstrates that RNA-Seq data can provide a comprehensive analysis of DT104 gene expression in a food model for poultry products. This study also provides additional evidence for the importance of metabolic adaptation in the ability of *S. enterica* to successfully adapt to and occupy niches outside of its host and provides potential targets that could be used to develop intervention strategies to control *Salmonella* in poultry.

## 1. Introduction

*Salmonella enterica* is one of the most important bacterial pathogens, causing over one million infections in the US each year [1]. In 2023, there were 1072 reported human illnesses caused by *Salmonella* associated with poultry, and 247 required hospitalizations [2]. Immunocompromised individuals, the elderly, and malnourished young children are particularly susceptible to infection with *Salmonella* associated with high mortality rates [3]. *S.* Typhimurium was responsible for a greater number of illnesses compared to *Salmonella enterica* Enteritidis (34% vs. 17.4%, respectively) in the years 1998 to 2008 [4].

Over 2600 *S. enterica* serovars have been identified, and all are considered pathogens; however, in the US, *S*. Enteritidis and *S.* Typhimurium are the most common serovars isolated from human foodborne infections [5,6]. In particular, the spread of *S.* Typhimurium definitive phage type 104 (DT104) has been a major concern for public health. DT104 developed multidrug resistance via the acquisition of *Salmonella* genomic island 1 and later spread globally [7]. DT104 is frequently isolated from human infections, poultry, and poultry products [8,9].

In multiple *S. enterica* outbreaks, raw material contaminating the processing plant environment has played a major role in the contamination of the food product [10]. This has led to studies investigating the adaptation, survival, and persistence of *S. enterica* serovars in food processing environments and food products. Goudeau et al. [11] used microarrays to measure the gene expression of *S.* Typhimurium in a model of lettuce and cilantro soft rot. They found that in this model, *S.* Typhimurium up-regulates genes for anaerobic metabolism and utilization of substrates, some of which were also up-regulated in multiple animal disease models [12,13]. In another complex food model, Deng et al. [14] used RNA-Seq to investigate the expression of genes involved in the long-term survival of *S.* Enteritidis in peanut oil. In their model, both heat and cold shock response genes were up-regulated with a low level of gene expression overall. Kjeldgaard et al. [15] used minced beef as a model for investigating gene expression in a cocktail of *S. enterica* strains. Their study showed that meat was a suitable model for measuring gene expression; however, it was limited to two stress response (heat and salt) genes and primarily served as a proof of concept. Differentially expressed genes were also identified using microarrays in *Listeria monocytogenes* grown in milk and ready-to-eat meat [16,17]. Despite the importance of poultry products, particularly raw or undercooked poultry, as a vehicle for *S. enterica* infections in humans, global gene expression in a poultry-product-based model remains poorly characterized in *S.* Typhimurium and other *S*. *enterica* serovars. Studies have shown that it is difficult to isolate RNA and perform transcriptomics from solid foods due to the complexity of the food matrix [18]. Fratamico et al. [19] developed a method using microarrays to study the gene expression of *E. coli* O157:H7 in ground beef extract (GBE) as a model that could be utilized to identify gene targets for interventions. Gene expression profiles of the pathogen exposed to GBE were compared to those in tryptic soy broth. Interestingly, *E. coli* O157:H7 exposed to GBE showed increased survival when subsequently exposed to synthetic gastric fluid at pH 1.5. Thus, a ground chicken extract (GCE) may be a better growth medium for mimicking a poultry environment in place of brain heart infusion (BHI) or other growth media for transcriptomic analysis of *Salmonella*.

*S.* Typhimurium DT104 was chosen for the focus of this study due to its relevance to public health and association with poultry products [8,9]. The aim of this study was to measure and characterize the global gene response of *S. *Typhimurium with exposure to a poultry-meat-based medium, i.e., GCE. Specifically, transcriptomic data were obtained and compared for DT104 in GCE and BHI medium. Overall, we found that differentially expressed genes (DEGs) showed significant enrichment for metabolic processes. The current study serves as proof of concept, and with further studies, the identified DEGs and pathways could serve as potential targets that can be employed to develop effective interventions for inactivating or preventing the growth of *Salmonella* in their natural food environment.

## 2. Materials and Methods

### 2.1. Bacterial Strains and Culture Conditions

*S.* Typhimurium strain ATCC 700408 (American Type Culture Collection, Manassas, VA, USA) multidrug-resistant, definitive phage type 104 (DT104) was used for this study and was maintained as previously described [20]. This DT104 strain is resistant to multiple antibiotics (chloramphenicol, ampicillin, tetracycline and streptomycin) [21]. *S.* Typhimurium DT104 is associated with poultry and the poultry environment, and strains of DT104 have been used in previous RNA-Seq studies [22]. One microliter of the stock culture was transferred into 5 mL of BHI and grown at 37 °C at 150 rpm to obtain cells in the early stationary phase (ESP, 24 h) as previously described [20]. 

### 2.2. RNA Isolation, Library Construction, and Sequencing

*S.* Typhimurium DT104 was cultured in BHI medium for 24 h to reach ESP. For RNA-Seq experiments, 1 mL of the starter culture from ESP was centrifuged for 5 min at 5000 rpm, and then the pellet was resuspended into 10 mL of BHI (control group) or GCE (experimental group) and adjusted to achieve a starting concentration of 10^4^ CFU/mL. GCE was prepared as previously described [20]. *S.* Typhimurium DT104 was grown at 37 °C at 150 rpm in BHI or GCE until the log phase (LP) was reached for optimal RNA quality. We had previously used plate counts to determine that 6.5 h of incubation was required for *S.* Typhimurium DT104 to reach the log phase for ESP cells [20]. Three independent samples were collected for each treatment (GCE vs. BHI). 

Extracted RNA was sequenced, and reads were mapped to the DT104 genome [22]. DEGs were identified in GCE when compared with those in BHI. Only genes that were significantly differentially expressed (≥2-fold and ≤−2 difference in expression with adjusted *p-*value < 0.05) were considered DEGs and selected for further analysis and discussion. 

RNA extraction was carried out by first centrifuging the cultures at 4000 rpm for 5 min and then immediately stabilizing them using RNA*later* RNA Stabilization Reagent provided in the QIAGEN RNeasy Midi Kit (QIAGEN Inc., Valencia, CA, USA). The total bacterial RNA was purified by using the AMBION TURBO DNA-*free*^TM^ Kit (Life Technologies Corporation, Carlsbad, CA, USA) according to the manufacturer’s instructions. The rRNA was removed using the Ribo-Zero Magnetic Kit for Gram-Negative Bacteria (Illumina Inc., San Diego, CA, USA), and then the RNA was concentrated and purified using RNA Clean & Concentrator (Zymo Research, Irvine, CA, USA). The RNA was quantified using a Nanodrop 1000 (Thermo Fisher Scientific, Waltham, MA, USA) and Qubit Fluorometric Quantitation (Thermo Fisher Scientific, Waltham, MA, USA). RNA quality was analyzed using an Agilent 2100 Bioanalyzer (Agilent Technologies, Santa Clara, CA, USA) according to the manufacturer’s protocol. The rRNA-depleted RNA was used for library construction using the TruSeq Stranded mRNA Library Prep Kit, and pair-end sequencing (2 × 75 nt) was performed in a MiSeq instrument using the 150-cycle V3 reagent kit (Illumina Inc., San Diego, CA, USA). Library preparation, cluster generation, and sequencing were performed according to the manufacturer’s recommendations (Illumina, Inc., San Diego, CA, USA).

### 2.3. RNA-Seq Data Analysis

RNA-Seq raw data were verified using the FastQC program (version 0.11.2). Subread package (version 1.6.2) was used to map sequencing reads to a fully sequenced *S.* Typhimurium DT104 strain [23]. The transcript levels were normalized using trimmed means M-values (TMM) from edgeR package (version 4.2.0). featureCounts [24] from subread package (version 1.6.2) was used to obtain the number of fragments per gene. The RNAseq read counts were normalized by using (Trimmed Mean of M-values) TMM in the edgeR package (version 4.2.0). R package limma (version 3.19) [25] was used to analyze differential gene expression. Differences greater than 2-fold (Log_2_FC greater than 1 or less than −1) and an adjusted *p*-value (FDR) < 0.05 were considered significant. Growth of the bacteria in BHI was used as the control group. PANTHER [26] classification system (version 17.0, http://pantherdb.org/) (accessed 1 December 2023) was further used for the GO (gene ontology) enrichment analysis of all the DEGs of *S.* Typhimurium DT104 in GCE. ggplot2 package (version 3.5.0) was utilized to visualize the results. 

### 2.4. Network Analysis

The top 10% up-regulated DEGs with the highest Log_2_FC value and the top 10% down-regulated DEGs with the lowest Log_2_FC value were used as the input genes to extract the corresponding protein–protein interaction (PPI) network. The STRING [27] database (version 11) was used to generate the PPI network of the input genes and the interactions of the corresponding proteins with a high confidence (cutoff score was 0.7). The score threshold of the STRING PPI network was from 0.15, which indicates low confidence, to 0.9, the highest confidence [28]. The PPI network was then clustered by Molecular Complex Detection (MCODE, version 2.0.2) using a two-degree cutoff and a haircutting option. The degree cutoff value is the setting that prevents nodes with fewer connections from being assessed and included in a cluster. The degree cutoff setting equals two, which allows MCODE to evaluate nodes with at least two interactions with other nodes. The highly interconnected proteins were clustered. Nonetheless, when a cluster of nodes was calculated and selected from the entire network, some nodes may have only one interaction with the cluster’s nodes. The haircut option would eliminate these types of nodes from the formed cluster. The node cutoff score was set as 0.2, which means the nodes have no less than 20% of the seed node score. Highly connected nodes and the corresponding interactions were obtained from the output of MCODE. The top three clusters were further analyzed using cytoHubba (version 0.1) [29]. Protein prioritization was evaluated through the Maximal Clique Centrality (MCC) algorithm in cytoHubba, and the top three prioritized proteins were selected.

## 3. Results and Discussion

### 3.1. RNA-Seq Data Analysis and Identification of DEGs in GCE

We were interested in identifying genes that were differentially expressed by *S.* Typhimurium DT104 in GCE. The DEGs in GCE compared to BHI medium were then identified through statistical analysis to measure and characterize the global gene response of DT104 in a poultry-meat-based medium (i.e., GCE). Although others have used media/extracts prepared with foods to investigate gene expression of pathogens in different types of foods [15,19], to our knowledge, our study is the first to use a poultry-meat-based medium for RNA-Seq analysis of *S*. Typhimurium DT104. On the basis of the identified targets (i.e., DEGs), GO enrichment, PANTHER pathway, and protein–protein interaction (PPI) analyses were conducted to pinpoint the biochemical pathways/processes involved in the adaptation and survival of DT104 in GCE.

The overall quality of RNA-Seq data is shown in Table 1, which demonstrates the total reads, percentage mapped, and percentage assigned for each sample. Three samples were collected for each treatment. All RNA-Seq samples have a percentage mapped score higher than 98.8% and a percentage assigned score higher than 90.1%. This dataset is of high quality or within the expected range for RNA-Seq data.

**Figure 1:**
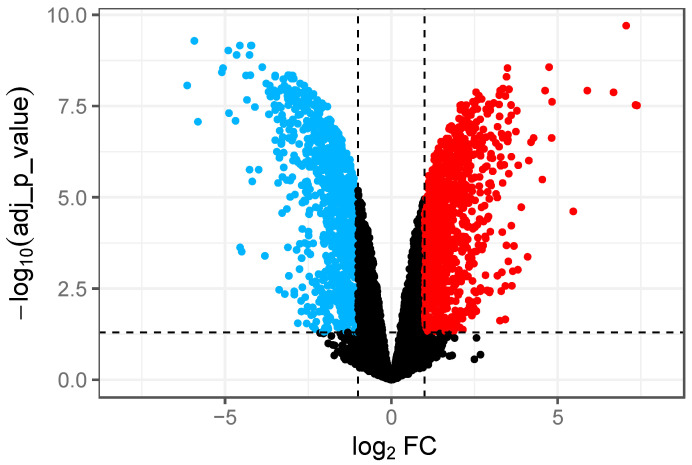
Volcano plot for comparison of DEGs between GCE and BHI. Red spots represent up-regulated genes with a threshold of Log_2_FC ≥ 1. Blue spots represent down-regulated genes with a threshold of Log_2_FC≤ −1. Both red and blue spots had an adjusted *p*-value < 0.05. Genes with no significant changes are indicated by black spots.

We observed 1690 DEGs which represented 36.7% of the genes in the DT104 genome. Of these, 902 were up-regulated in GCE while 788 genes were down-regulated (Supplemental Table S1). Volcano plots were used to represent variances in DEGs (Figure 1). Specifically, 902 up-regulated genes (shown in RED in Figure 1) and 788 down-regulated genes (shown in BLUE in Figure 1) were identified in GCE. The DEGs with high and low fold changes are located at the top-left and top-right corners, respectively, in Figure 1. In addition, the top twenty genes with the highest fold changes are listed in Table 2 and Table 3 for the up-regulated and down-regulated genes, respectively. 

**Figure 2:**
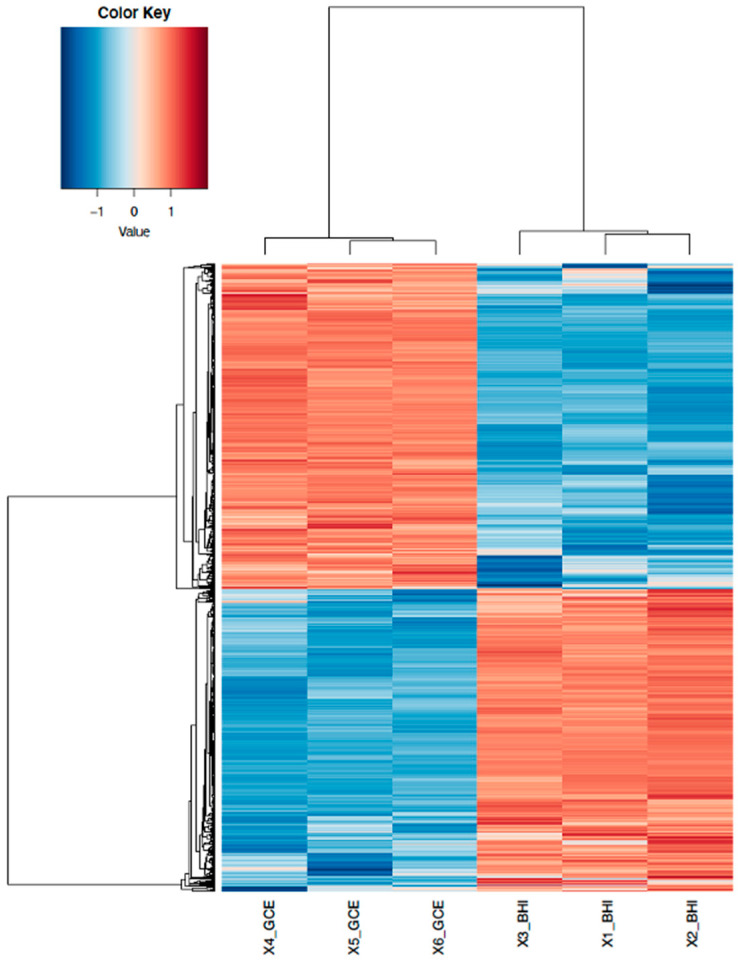
Heatmap of the DEGs’ response to GCE and BHI in *Salmonella* DT104. DEGs with a threshold of Log_2_FC ≥ 1 are shown in red, whereas DEGs with a threshold of Log_2_FC ≤ −1 are shown in blue.

DEGs were further classified into different groups in which DEGs showing similar expression patterns were clustered into the same group in the heatmap trees (Figure 2). While little variation existed in the three samples for the same type of treatment (e.g., BHI or GCE), the expression patterns of DEGs were similar among treatment replicates, indicating reproducibility between the two treatments for the RNA-Seq experiments.

### 3.2. GO and PANTHER Pathway Analysis of DEGs in GCE

**Figure 3:**
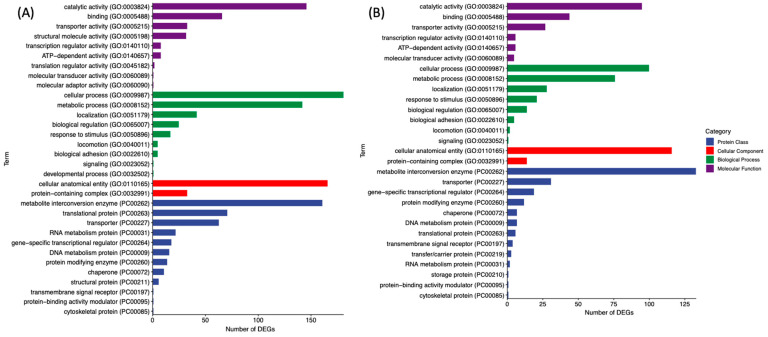
GO enrichment analysis: (**A**) up-regulated DEGs in GCE; (**B**) down-regulated DEGs in GCE.

The DEGs in Figure 1 and Figure 2 were further analyzed through the GO analysis for gene function in terms of molecular function (purple), biological process (green), cellular component (red) and protein class (blue). There were 20 and 16 categories differentially enriched for up-regulated and down-regulated genes, respectively (Figure 3). For molecular function, the up-regulated DEGs had additional translation regulator and molecular adaptor activities compared to down-regulated DEGs. For biological processes, up-regulated DEGs had additional responses to stimulus, biological adhesion, and developmental process activities compared to down-regulated DEGs. As for protein class (blue), there were 12 and 13 categories of proteins encoded by up-regulated and down-regulated DEGs, respectively. More up-regulated DEGs were involved in encoding translational proteins and RNA metabolism proteins (Figure 3). 

**Figure 4:**
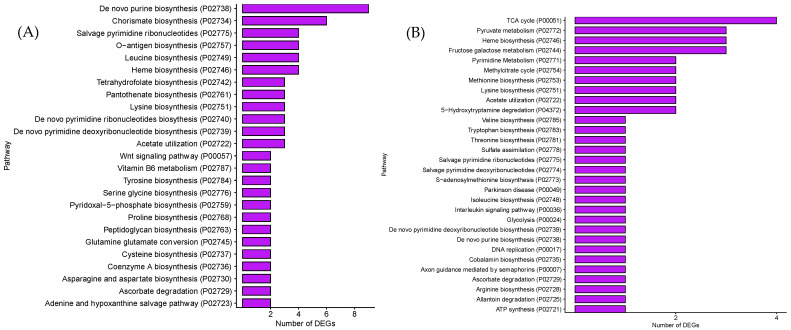
PANTHER pathway analysis: (**A**) up-regulated DEGs in GCE; (**B**) down-regulated DEGs in GCE.

The pathways for the involvement of these DEGs were further analyzed via PANTHER pathway analysis (Figure 4). As for the up-regulated DEGs, the major pathways that had more than three DEGs included the following: biosynthesis of De novo purine, Chorismate, Salvage pyrimidine ribonucleotides, O-antigen, Leucine, Heme, Tetrahydrofolate, Pantothenate, Lysine, De novo pyrimidine ribonucleotides, De novo pyrimidine deoxyribonucleotide, and Acetate utilization. Only four pathways, including TCA cycle, Pyruvate metabolism, Heme biosynthesis, and Fructose galactose metabolism, had more than three down-regulated DEGs. 

From the GO enrichment analysis and PANTHER pathway analysis, we noticed the complex modulation of various genes through the identification of both up-regulated and down-regulated genes within key pathways during GO enrichment analysis. For example, in Figure 3, catalytic activity proteins gathered the highest number of up-regulated genes and also gathered the highest number of down-regulated genes. Due to the complex gene regulation in *Salmonella* Typhimurium DT104, analyzing individual gene expression levels alone may not provide enough information about the functional consequences or the interactive effects of these genes in the cellular context [30].

### 3.3. PPI Network and Clusters of DEGs

**Figure 5:**
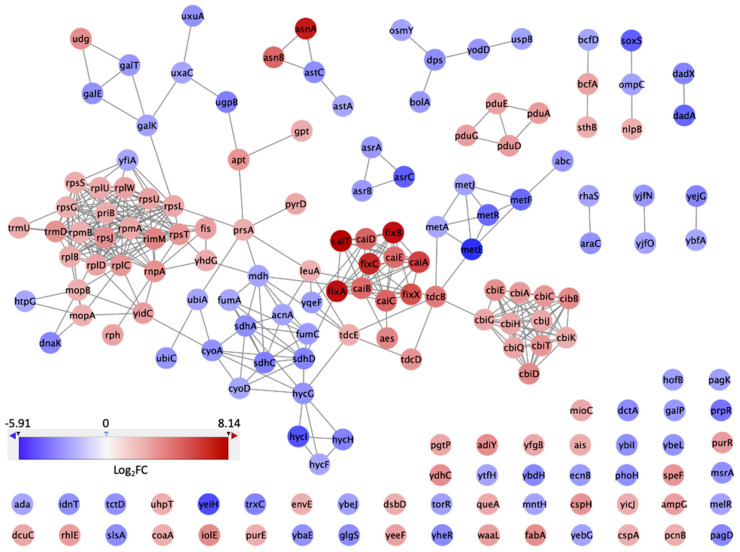
Protein-protein interaction network of top 10% over-expressed genes (nodes in red color with a threshold of Log_2_FC ≥ 1) and top 10% repressed genes (nodes in blue color with a threshold of Log_2_FC < 1) in GCE.

**Figure 6:**
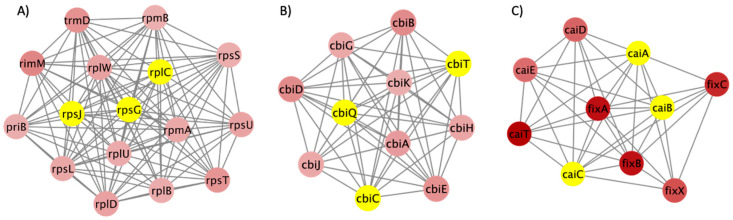
Top three clusters generated by MCODE. (**A**) Cluster 1 with MCODE score 14.933; (**B**) Cluster 2 with MCODE score 11; (**C**) Cluster 3 with MCODE score 8.667. Proteins are represented by nodes. Red nodes represent proteins with a threshold of Log_2_FC ≥1. Yellow nodes are the hub proteins extracted by cytoHubba.

In order to tackle the issue of the complex regulation of various genes, we expanded our analysis to incorporate protein–protein interaction (PPI) network analysis. Performing PPI network analysis enables us to visually represent and understand the functional connections between proteins encoded by the genes expressed differentially [31]. This approach is especially beneficial in elucidating intricate biological processes and comprehending the role of specific pathways in the bacterium’s ability to flourish in GCE. Our goal was to analyze these interactions to identify important protein hubs and pathways crucial for the survival and virulence of *S.* Typhimurium DT104 in the specific conditions being studied. The PPI network of the top 10% DEGs in GCE is shown in Figure 5. In this network, there were 168 nodes, which represented 168 proteins encoded by the genes from the input DEGs. There were 355 interactions between the 168 proteins shown as the edges in Figure 5. Different colors represent different Log_2_FC values, with blue corresponding to lower Log_2_FC values and red corresponding to higher Log_2_FC values. There were 10 clusters generated by MCODE, with scores ranging from 14.933 to 3.000. The score of each cluster in MCODE indicates the density of connections between the proteins within each node. Accordingly, higher scores indicated more interconnected nodes. The top three clusters with the highest scores are shown in Figure 6. Figure 6A shows cluster 1 with 16 ribosomal proteins and their 112 interconnections with a score of 14.933. Figure 6B shows cluster 2 with 11 proteins involved in vitamin B12 biosynthesis and their 55 interconnections with a score of 11. Figure 6C shows cluster 3 with 10 proteins involved in carnitine metabolism and their 39 interconnections with a score of 8.667. The nodes with yellow color are the top three hub proteins of each cluster extracted by cytoHubba. Proteins RpsJ, RpsC, RpsG in cluster 1; CbiQ, CbiC, CbiT in cluster 2; and CaiA, CaiB, CaiC in cluster 3 had the most connections within the clusters. The high importance of ribosomal proteins (RpsJ, RpsC, RpsG) in cluster 1 highlights the increased requirements for protein synthesis as the bacterium adjusts to and multiplies within the GCE environment [32]. The proteins CbiQ, CbiC, and CbiT play a role in the transport and synthesis of cobalamin (vitamin B12). This suggests that they are part of an adaptive response to ensure that there is enough cofactor available, which is essential for many enzymatic processes [33]. Cluster 3 exhibits the presence of CaiA, CaiB, and CaiC, which play a role in carnitine metabolism. This suggests that there is a metabolic adjustment that enables the efficient use of nutrients for energy production and stress responses.

### 3.4. Comparative Analysis of DEGs That Were Expressed in Response to GCE

A nutrient-rich broth commonly used for the growth of *S.* Typhimurium and other fastidious and non-fastidious bacteria is BHI, which includes (g/L) brain heart, infusion from solids (6), peptic digest of animal tissue (6), pancreatic digest of gelatin (14.5), dextrose (3), sodium chloride (5), and disodium phosphate (2.5). The components of chicken meat are as follows: proteins and all of the essential amino acids, minerals, including calcium, iron, magnesium, phosphorous, potassium, sodium, zinc, and selenium, and vitamins, including niacin, pantothenic acid, vitamins B6 and B12, folate, and folic acid [34]. BHI has 3.0 g/L of dextrose, which is not found in GCE, and GCE has a pH of 6.4 as opposed to BHI, which has a pH of 7.4. GCE does not contain carbohydrates, while BHI contains dextrose. In a previous study, Hawkins et al. [20] showed that overall, DT104 grown in GCE had a somewhat lower Y_max_ (maximum CFU/mL reached under certain conditions) than in BHI (log_10_ 8.77 vs. 9.12, respectively), possibly due to limited nutrients in GCE. 

Comparing GCE to BHI following growth to the ESP, we observed top up-regulated DEGs enriched for carnitine metabolism (Table 2). A closer look at the gene expression in enriched processes revealed that six (*caiA-E* and *caiT*) carnitine metabolism genes showed the greatest fold change with a 23.3- to 282.8-fold increase in expression for DT104 grown in GCE compared to BHI (Table 2). In addition, the *fixA-C* and *fixX* genes were also up-regulated in DT104 in GCE. The *fixA* and *fixB* genes have been shown to be involved in carnitine reduction in *E. coli* [35]. One of the potentially abundant substrates available to DT104 in GCE is carnitine. GCE has enriched carnitine also due to its application as a feed additive for poultry [36,37]. Carnitine is a quaternary ammonium compound involved in the metabolism of fatty acids. In mammals and birds, it is found in high concentrations in skeletal muscle. In chicken breast, the concentration is ~35 µg/g [38]. Carnitine metabolism pathways were found to be up-regulated in GCE compared to BHI following ESP. This may be due to the limited nutrients in GCE, containing no carbohydrates, while BHI contains dextrose. Carnitine might be used as a carbon, nitrogen, and energy source for DT104’s survival in GCE; however, this requires additional studies. This is consistent with the fact that carnitine can be used as a sole carbon and nitrogen source by bacteria, as well as a compatible solute to survive stress [39,40]. The up-regulation of these genes facilitates the adaptation of *Salmonella* to available nutritional sources. A previous study has shown that the reduction of the carnitine molecule is associated with increased growth of *S.* Typhimurium [41]. 

In addition to the differences in nutrient availability in GCE and BHI, pH is another environmental factor to which DT104 must be able to adapt. We had previously observed an acidic pH in the GCE medium (GCE has a pH of 6.4 compared to 7.4 for BHI) [20]. To counteract a low pH, *Salmonella* has inducible amino acid decarboxylase genes, which have been shown to raise the pH in the surrounding environment [40]. The ornithine decarboxylase gene, *speF*, was induced (9.6-fold) in ESP-GCE compared to ESP-BHI (Supplemental Table S1). Of the inducible amino acid decarboxylases in *S.* Typhimurium, *speF* was shown to be the best at promoting growth under low oxygen conditions [42]. 

One of the biological processes up-regulated in the ESP in GCE compared to the BHI medium was the synthesis of cobalamin. This molecule is also known as vitamin B_12_, and in bacteria, it is thought to assist with the metabolism of small molecules. In *S.* Typhimurium, the use of cobalamin for utilization of ethanolamine and propanediol is well documented [43,44]. These molecules are thought to be important nutrient sources in the inflamed gut of hosts and provide *Salmonella* a competitive advantage over the resident microflora [13,33,43,45]. The importance of these molecules in meat products for possibly providing *Salmonella* an advantage in the meat processing environment has not been documented to our knowledge. Other researchers have observed that other foodborne pathogens likely use these molecules in food processing environments. Specifically, Tang et al. [46] found that cobalamin biosynthesis, ethanolamine metabolism, and propanediol metabolism genes were up-regulated in *L. monocytogenes* when grown on salmon.

The network analysis of this study also investigated the DEGs at a systematic level and helped to unveil the potential mechanism utilized by DT104 to achieve survival in GCE. Ribosomal proteins, cobalamin biosynthesis proteins, and carnitine metabolism proteins were the proteins in the highly scored clusters. Ribosomal proteins in cluster 1 were over-expressed in *S.* Typhimurium DT104 in GCE. This is consistent with previous research that indicated that ribosomal genes were up-regulated in *S. enterica* exposed to contaminated produce [47]. An overall increase in cellular protein biosynthesis was deduced from the significant up-regulation of ribosomal subunit proteins. Cobalamin biosynthesis proteins in cluster 2 were over-expressed. Cobalamin is a cofactor for ethanolamine breakdown, which is essential for *S.* Typhimurium DT104. Carnitine metabolism proteins in cluster 3 were over-expressed because carnitine could be used as a carbon and nitrogen source for *S.* Typhimurium DT104. Hub proteins encoded by the genes *rpsJ*, *rpsC*, *rpsG*, *cbiQ*, *cbiC*, *cbiT*, *caiA*, *caiB*, and *caiC* could be utilized as potential targets for growth inhibition.

## 4. Conclusions

The current study serves as a proof of concept showing that a poultry-meat-based medium can be used as a model food system to study gene expression of *Salmonella* as opposed to a commonly used growth medium such as BHI. It provides insights into the transcriptomic response of DT104 with exposure/growth in a poultry-meat-based medium. Overall, we found that differentially expressed genes (DEGs) showed significant enrichment for metabolic processes, including genes for carnitine metabolism. The current study also provides baseline data for identifying molecular targets for intervention strategies to control *S.* Typhimurium DT104 in poultry products. One of the potential challenges with identifying metabolic targets for such interventions is that there is a broad range of metabolic pathways available to *Salmonella* in the absence of preferred substrates. Studies are needed to understand how to inhibit the expression of the potential target genes and, therefore, reduce the growth of *Salmonella* in poultry and the poultry processing environment. The results of this study show the utility of GCE as a model system for *Salmonella* gene expression studies in poultry and poultry products.

## Figures and Tables

**Table 1:** Overall quality of RNA-Seq data.

Sample Name	Total Reads	Percentage Mapped	Percentage Assigned
X1-BHI	2,519,286	99.7	92.6
X2-BHI	2,631,410	99.7	93.8
X3-BHI	2,336,974	98.1	93.3
X4-GCE	2,565,122	98.8	93
X5-GCE	3,093,996	99.6	90.1
X6-GCE	3,298,803	99.7	92.4

**Table 2:** Top twenty up-regulated DEGs identified in GCE.

Gene Locus	Gene Function	Log_2_FC	Fold Change
*caiT*	Probable carnitine transporter	8.14	282.80
*fixA*	FixA protein	7.38	167.15
*fixB*	FixB protein	7.34	162.02
*asnA*	Asparagine synthetase A	7.06	133.51
*fixC*	FixC protein	6.68	102.42
*caiA*	Probable carnitine operon oxidoreductase CaiA	5.89	59.29
*fixX*	Ferredoxin like protein FixX	5.47	44.26
*caiC*	Probable crotonobetaine/carnitine-CoA ligase	4.83	28.42
*caiD*	Carnitine racemase	4.82	28.21
*asnB*	Asparagine synthetase B	4.74	26.77
*caiB*	L-carnitine dehydratase	4.62	24.65
*caiE*	Carnitine operon protein CaiE	4.54	23.25
*tdcB*	Catabolic threonine dehydratase	4.28	19.41
*pefB*	Plasmid-encoded fimbriae regulation	4.20	18.35
*rim*	16S rRNA processing protein RimM	4.13	17.56
*aes*	Acetyl esterase	4.10	17.09
*gtrB*	Glycosyltransferase	3.90	14.97
*iolE*	Inosose dehydratase ec= altname: full = 2-keto-myo-inositol dehydratase	3.82	14.08
*cbiB*	Cobalamin biosynthesis protein	3.80	13.97
*cbiD*	Putative cobalt-precorrin-6A synthase	3.77	13.67

**Table 3:** Top twenty down-regulated DEGs identified in GCE.

Gene Locus	Gene Function	Log_2_FC	Fold Change
*hslT*	Heat shock protein A	−6.13	−70.0
*metE*	5-methyltetrahydropteroyltriglutamate-homocysteine methyltransferase	−5.92	−60.6
*hslS*	Heat shock protein B	−5.81	−56.1
*yeiH*	Putative membrane protein	−5.09	−34.1
*ybiY*	Putative pyruvate formate-lyase 3 activating enzyme	−5.06	−33.4
*hycI*	Hydrogenase 3 maturation protease	−4.90	−29.9
*ybiW*	Putative formate acetyltransferase 3	−4.65	−25.1
*asrC*	Anaerobic sulfite reductase subunit C	−4.55	−23.4
*soxS*	Regulatory protein SoxS	−4.54	−23.3
*dadA*	D-amino acid dehydrogenase small subunit	−4.37	−20.7
*metR*	Trans-activator of MetE and MetH	−4.24	−18.9
*metF*	5,10 methylenetetrahydrofolate reductase	−4.22	−18.6
*prpR*	Propionate catabolism operon regulatory protein	−3.99	−15.9
*exuT*	Hexuronate transporter	−3.88	−14.7
*sdhC*	Succinate dehydrogenase cytochrome b-556 subunit	−3.65	−12.6
*ugpB*	Glycerol-3-phosphate-binding periplasmic protein	−3.63	−12.4
*hycH*	Formate hydrogenlyase maturation protein	−3.52	−11.5
*dadX*	Alanine racemase	−3.50	−11.3
*trxC*	Thioredoxin 2	−3.49	−11.2
*sdhD*	Succinate dehydrogenase hydrophobic membrane anchor protein	−3.45	−10.9

## Data Availability

Raw sequencing data were deposited into the NCBI database under BioProject ID PRJNA987661.

## Supplementary Materials

The following supporting information can be downloaded at https://www.mdpi.com/article/10.3390/microorganisms12071461/s1: Table S1: DEGs of *S.* Typhimurium DT104 in GCE.
